# Effects of Autogenic Training on Pain Modulation in Burning Mouth Syndrome: A Preliminary Study

**DOI:** 10.7759/cureus.80549

**Published:** 2025-03-13

**Authors:** Keita Takizawa, Kana Ozasa, Kohei Shimizu, Noboru Noma

**Affiliations:** 1 Department of Oral Medicine, Nihon University School of Dentistry, Tokyo, JPN; 2 Department of Endodontics, Nihon University School of Dentistry, Tokyo, JPN

**Keywords:** autogenic training, burning mouth syndrome, conditioned pain modulation, emotional regulation, temporal summation

## Abstract

Introduction and aim: Burning mouth syndrome (BMS) is a chronic pain condition lasting more than 3-6 months. While various pharmacological treatments are used, no definitive treatment exists. Autogenic training (AT), a relaxation technique, helps manage stress-related pain by influencing brain regions involved in emotion regulation and cognitive control. Reduced conditioned pain modulation (CPM) efficiency occurs in chronic pain conditions, and in BMS, higher state anxiety negatively impacts the descending pain modulation system. This study aimed to evaluate the effect of AT on spontaneous pain reduction in patients with BMS and determine if AT improves CPM, particularly in those with chronic BMS.

Methods: This study included 28 patients diagnosed with BMS, along with 17 healthy volunteers. Based on the duration the patients experienced the pain, those with BMS were categorized into subchronic (≤6 months) and chronic (>6 months) groups. All participants’ temporal summation of pain (TSP), CPM, and pain intensity were recorded before and after the AT intervention. TSP was assessed through repeated electrical stimulation to the chin and was calculated as the difference between the visual analog scale scores after 10 electrical stimuli and the score following the first stimulus. CPM was calculated as the difference in TSP at baseline and following the conditioning of painful (47°C) or non-painful (40°C) stimuli applied to the non-dominant hand, serving as the conditioning stimulus (CS). This study was approved by the Ethical Committee of the Nihon University School of Dentistry (EP21D002) and conducted in accordance with the Declaration of Helsinki.

Results: AT significantly reduced spontaneous pain in the chronic BMS group but not in the subchronic group. Furthermore, CPM improved only in patients with chronic BMS during painful stimuli, suggesting enhanced pain modulation. Correlation analysis between BMS duration and CPM revealed a negative correlation between painful CS CPM and disease duration (r = -0.411, p < 0.05), but no correlation when the CS was not painful.

Conclusion: AT can reduce pain in patients with chronic BMS (lasting more than six months) at least partly by enhancing the patients’ pain modulation and emotional regulation, making it a potential adjunctive therapy for chronic cases.

## Introduction

The International Classification of Headache Disorders (ICHD)-3 offers a comprehensive framework for categorizing burning mouth syndrome (BMS) [1]. BMS is often classified as acute or chronic types based on its duration [2]. There is no gold standard to define pain as chronic; however, pain persisting or recurring for >3-6 months is considered a chronic condition [3]. Burning mouth, in contrast, is distinct from BMS, as it is secondary to underlying local or systemic factors, and addressing these factors is the primary approach to treatment. On the other hand, BMS is idiopathic with probable neuropathic causes, including chorda tympani dysfunction, and is primarily managed through pharmacotherapy, psychotherapy such as cognitive behavioral therapy (CBT), and other therapeutic modalities [4-6].

Pharmacotherapy is the primary treatment for BMS, with clonazepam, capsaicin, and alpha-lipoic acid among the most commonly used drugs [4,5]. Despite findings from relatively small randomized controlled trials, no definitive treatment has been established [6]. In addition to pharmacotherapy, non-drug treatment options, such as psychological therapies, have been shown to improve pain and oral health-related quality of life in patients with BMS [7].

Autogenic training (AT) is a self-suggestive-based relaxation method designed to teach patients how to control their stress response or, at minimum, recognize stress triggers and respond adaptively [8]. AT has shown efficacy in managing stress-influenced pain conditions like migraines [9]. Its effectiveness is attributed to the fact that the stress response is mediated by the neuroendocrine system, particularly through the hypothalamic-pituitary-adrenal axis, which is regulated by a negative feedback mechanism and a network of brain areas [10]. AT reportedly modulates the brain regions responsible for emotion recognition, emotional integration, and cognitive control, thereby reducing the frequency and severity of migraine attacks [9].

Pain modulation is commonly studied using the conditioned pain modulation (CPM) experimental paradigm. Temporal summation of pain (TSP) evaluates the facilitatory modulation process by assessing changes in pain perception in response to a series of repeated, consistent noxious stimuli. In contrast, CPM represents the inhibitory modulation process, indicating the effectiveness of endogenous analgesia mediated by the descending pain-modulatory system [11,12]. Low CPM efficiency has been observed in individuals with chronic pain and has also been identified as a predictive factor for the onset of chronic pain in those at risk [11]. In a previous study, we found a significantly positive correlation between CPM and both state and trait anxiety in patients with BMS, suggesting that higher levels of state anxiety negatively affect the descending pain modulation system [13]. Specifically, higher state anxiety reduced the CPM effect induced by noxious conditioning stimuli (CS).

This preliminary study hypothesized that AT improves CPM efficiency and reduces spontaneous pain in BMS patients.

## Materials and methods

The study was conducted at Nihon University Dental Hospital, Japan, with approval from the Ethical Committee of the Nihon University School of Dentistry (EP21D002), and in accordance with the Declaration of Helsinki. All participants provided written informed consent. The examiner, blinded to participant status, conducted all TSP and CPM examinations before and after AT. This study included 28 females with BMS and 17 healthy female volunteers. Patients with BMS were categorized into two groups based on the duration of their condition as follows: ≤6 months (subchronic BMS, n = 13) and >6 months (chronic BMS, n = 15). The control group comprised 17 healthy volunteers recruited from the staff of a dental hospital. BMS was diagnosed according to the ICHD-3 criteria. The inclusion criteria for patients with BMS included superficial intraoral pain lasting >3 months, persistent burning pain (>2 hours/day), and no visible clinical changes in the oral mucosa (e.g., redness, swelling, lichen planus, or ulcers) [1,12]. Each participant was examined in a laboratory setting and exposed to the following two psychophysical tests: TSP and CPM. No significant age difference was observed among patients with BMS (60.9 ± 13.5 years) and controls (56.8 ± 11.7 years) or between subchronic (58.3 ± 12.8 years) and chronic patients with BMS (63.2 ± 14.3 years).

Temporal summation of pain

To assess TSP, an intradermal electrical stimulation was administered to the right chin using a stainless-steel concentric bipolar electrode (Nihon Kohden, Tokyo, Japan) as described by Inui et al. [14]. The electrode consisted of a cylindrical anode (diameter: 1.4 mm) encircling a centrally placed pushpin-shaped cathode (diameter: 0.2 mm). The pin cathode was designed to protrude at 0.1 mm from the outer ring anode, allowing the tip to be inserted into the epidermis without causing discomfort when pressed against the skin. The electrode was positioned on the chin at the midpoint between the mouth angle and midface.

The intensity of TSP stimulation was set at the pain threshold for a single stimulus. After one electrical stimulus, 10 consecutive stimuli were delivered at a frequency of 1 Hz. Participants rated their pain intensity using the Numerical Pain Scale (NPS), with scores recorded after both the single and 10 consecutive stimuli [12]. TSP was calculated as the difference between the NPS scores after 10 electrical stimuli and the NPS score after one electrical stimulus.

Conditioned pain modulation

For the CPM assessment, a thermode (Intercross 210, Tokyo, Japan) was used to apply either non-painful (40°C) or painful (47°C) stimulation to the participant's non-dominant hand for 10 seconds, serving as the CS. The thermode featured a Peltier element with a 10 × 10 mm contact area. Participants rated the pain intensity using the NPS. The subjects underwent TSP at baseline and following exposure to the CS. The washout period between the two sessions was one hour. The difference between TSP without CS and TSP with non-painful or painful CS was considered as the CPM score. Negative values indicated significant pain reduction, representing effective CPM [12].

The autogenic training

**Figure 1:**
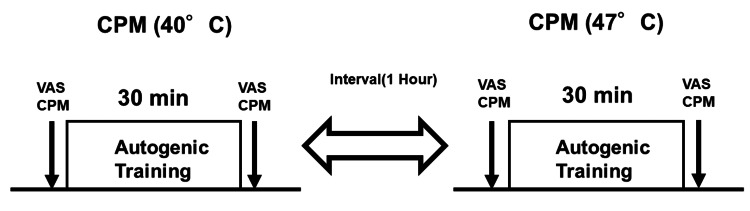
Illustration of the experimental procedure in each session First, CPM (40°C) was measured before and after AT, followed by a one-hour break, after which CPM (47°C) was measured the same way. For subchronic and chronic BMS groups, visual analog scale (VAS) scores (spontaneous pain) were measured before and after AT. CPM: conditioned pain modulation; AT: autogenic training; BMS: burning mouth syndrome; VAS: visual analog scale

The AT intervention included the following six standard exercises: (1) limb heaviness, (2) limb warmth, (3) cardiac, (4) respiration, (5) solar plexus warmth, and (6) "forehead cooling" exercises [15]. In this study, the AT session using standard exercise 1 based on the Schultz method lasted approximately 30 minutes and was facilitated by a prerecorded AT tape [16]. In brief, the first standard exercise of AT involves several steps aimed at inducing relaxation. Participants begin by assuming a comfortable position and closing their eyes to focus on deep, rhythmic breathing. They then mentally repeat suggestions of heaviness for each limb, such as “My arm is heavy.” After establishing a sense of heaviness, they affirm, “My whole body is heavy,” visualizing their body sinking into the surface beneath them. The session concludes with a gradual return to awareness, including deep breathing and gentle stretching, promoting an enhanced state of relaxation and well-being [16]. The first 15 minutes of the study involved instructing the subjects on relaxation techniques, such as abdominal breathing, to promote relaxation, with breathing and relaxation exercises performed twice daily, each lasting 15 minutes, on the day of the study. In the final 15 minutes, the patients were instructed to continue standard exercise 1 independently while listening to a prerecorded AT tape. All participants completed two CPM sessions at two different temperatures (40°C and 47°C) at approximately the same time of day, with a minimum interval of one hour between each session (Figure 1).

Statistical analysis

For statistical analysis, the Shapiro-Wilk test revealed that none of the comparisons followed a normal distribution; therefore, nonparametric methods were applied. The Wilcoxon signed-rank test was used to examine changes in VAS and CPM scores pre- and post-AT sessions. TSP and CPM were analyzed using the Kruskal-Wallis test, followed by the Steel-Dwass post hoc correction for multiple group comparisons. Spearman’s rank test was used to assess the correlations between variables, including VAS, disease duration, TSP, and CPM effect. CPM efficiency was calculated as the percentage change in the CPM effect, defined as (post-CPM TSP - pre-CPM TSP) × 100%. The CPM effect was determined using the following formula: (TSP with condition - TSP) / TS × 100%. All analyses were conducted using IBM SPSS Statistics for Windows, Version 20 (Released 2011; IBM Corp., Armonk, New York, United States). Statistical significance was set at p < 0.05. Before the initiation of this study, statistical power analysis was conducted using G*Power (version 3.1, Heinrich-Heine-Universität Düsseldorf, Düsseldorf, Germany). For statistical analysis, the study samples were classified into two or three groups. The calculations indicated that a minimum sample size of 53 for comparisons between two groups and 14 for comparisons among three groups was required to achieve 80% statistical power with a significance level of 0.05 and an effect size of 0.5. However, as this study is a preliminary experiment, an interim stage was set, and a minimum sample size of 13 and 15 for two- and three-group comparisons, respectively, was used for exploratory statistical analysis.

## Results

**Figure 2:**
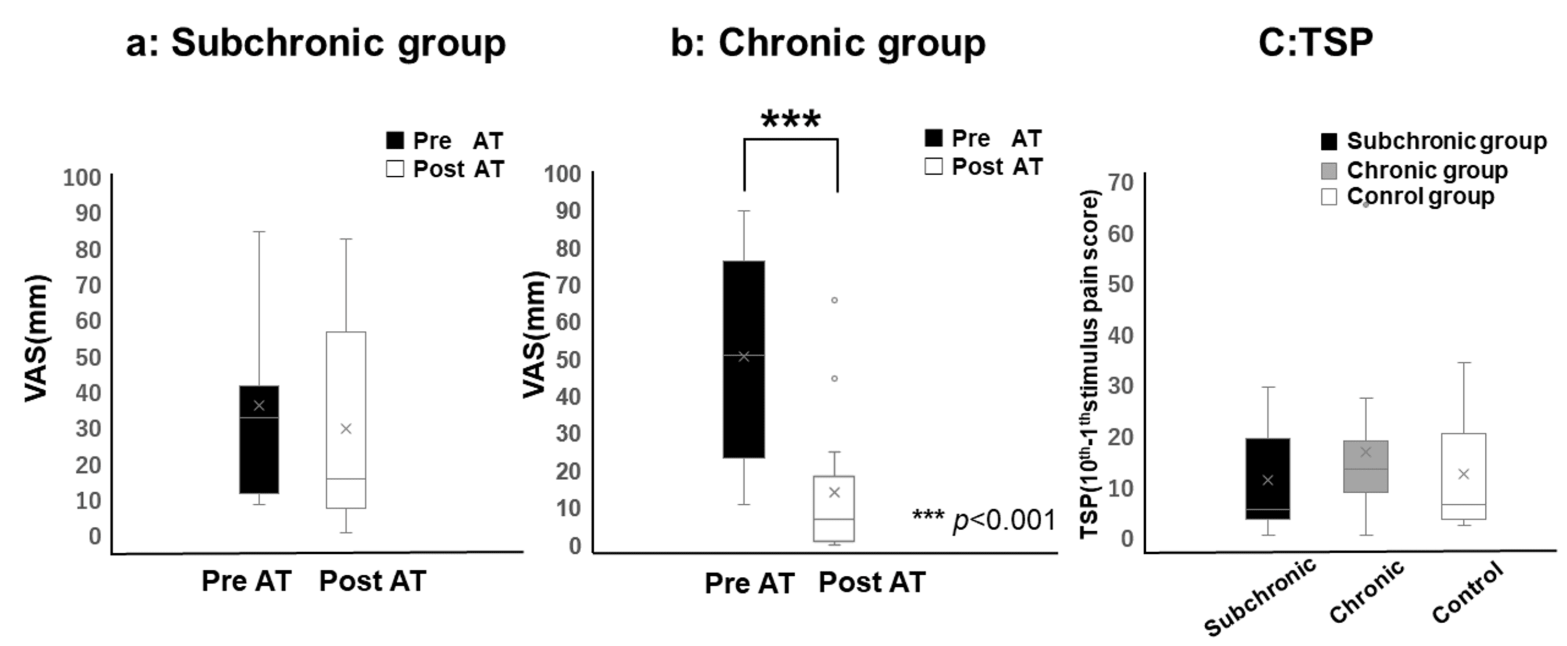
Changes in spontaneous pain before and after AT (a) In the subchronic group, no significant changes in pain were observed before and after AT, (b) while in the chronic group, a significant reduction in pain was noted after AT (p < 0.001). (c) No significant differences in TSP values were found across the subchronic BMS, chronic BMS, and control groups before AT. In the box-and-whisker plot, whiskers extend to 1.5 times the interquartile range, with points beyond this range marked as outliers. AT: autogenic training; BMS: burning mouth syndrome; TSP: temporal summation of pain; VAS: visual analog scale

The overall pain intensity (measured by VAS) decreased by an average of approximately 17.79% in the subchronic BMS group and 72.12% in the chronic BMS group after the application of AT. The chronic BMS group demonstrated a significant reduction in pain post-AT intervention (p < 0.001). In the subchronic BMS group, the mean VAS scores were 32.0 (range: 11.0-41.0) and 15.0 (range: 7.0-56.0) pre- and post-AT, respectively. In the chronic BMS group, the mean VAS scores were 51.0 (range: 23.5-76.5) and 7.0 (1.0-18.5) pre- and post-AT, respectively (Figure 2).

In the subchronic BMS group, the average NPS score for a single pulse applied to the chin was 20.0 (range: 19.0-24.0), increasing to 26.0 (range: 21.0-45.0) after 10 pulses. In the chronic BMS group, the mean NPS score for a single pulse on the chin was 20.0 (range: 20.0-24.5), increasing to 33.0 (range: 29.0-44.5) after 10 pulses. In the control group, the average NPS score for a single pulse on the chin was 6.0 (range: 4.0-9.0), increasing to 15.0 (range: 9.0-26.0) after 10 pulses. No significant differences were observed in TSP values between groups before AT. TSP values in the subchronic BMS, chronic BMS, and control groups were 5.0 (range: 3.0-19.0), 13.0 (range: 8.5-18.5), and 6.0 (range: 3.0-20.0), respectively (Table 1).

**Table 1:** Participant demographics and pain modulation values before and after autogenic training Participant demographics and values of TSP and CPM in the painful session (47°C) before and after AT in the subchronic, chronic, and control groups. Data are presented as means and standard deviations for age and as medians and interquartile ranges for TSP and CPM. TSP: temporal summation of pain; CPM: conditioned pain modulation; AT: autogenic training

Group	Pre-AT TSP	Pre-AT CPM	Post-AT TSP	Post-AT CPM
Subchronic BMS (n = 13, female, 58.3 ± 12.8 years)	5.0 (3.0-19.0)	0.0 (-7.0 to 4.0)	15.0 (3.0-32.0)	-1.0 (-11.0 to 0.0)
Chronic BMS (n = 15, female, 63.2 ± 14.3 years)	13.0 (8.5-18.5)	-1.0 (-7.0 to 5.0)	10.0 (7.0-25.0)	-4.0 (-12.5 to -1.5)
Control (n = 17, female, 56.8 ± 11.7 years)	6.0 (3.0-20.0)	-2.0 (-5.0 to 2.0)	3.0 (2.0-6.0)	0.0 (-1.0 to 2.0)

**Figure 3:**
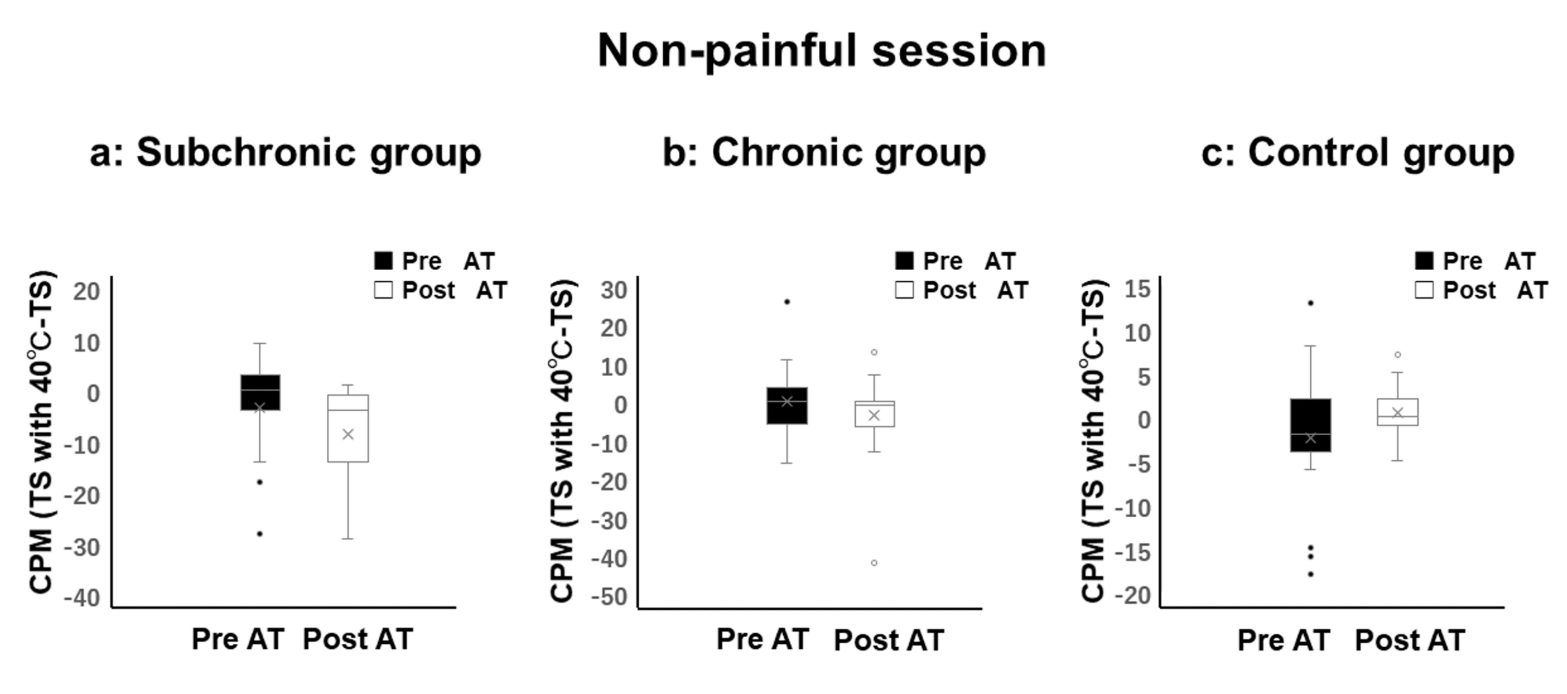
Evaluation of conditioned pain modulation (CPM) during the non-painful session (40°C) No significant improvements in CPM were observed before and after AT in the subchronic, chronic, or control groups. In the box-and-whisker plot, whiskers extend to 1.5 times the interquartile range, with points beyond this range shown as outliers. CPM: conditioned pain modulation; AT: autogenic training; TS: temporal summation

No significant differences were observed in CPM values among the subchronic BMS, chronic BMS, and control groups prior to AT. No significant differences were observed in CPM following the non-painful CS values before and after AT among the subchronic BMS group (pre-AT = 0.0 (range: -4.0 to 3.0); post-AT = -4.0 (range: -14.0 to -1.0)), chronic BMS group (pre-AT = 0.0 (range: -6.0 to 3.5); post-AT = -1.0 (range: -6.5 to 0.0)), and control group (pre-AT = -2.0 (range: -4.0 to 2.0), post-AT = 0.0 (range: -1.0 to 2.0) (Figure 3).

**Figure 4:**
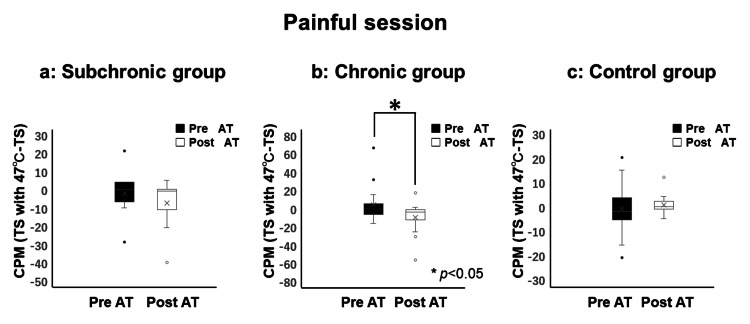
Evaluation of conditioned pain modulation (CPM) during the painful session (47°C) No significant differences were observed before and after AT in the subchronic and control groups, but the chronic group showed significant improvement in CPM after AT (p < 0.05). In the box-and-whisker plot, whiskers extend to 1.5 times the interquartile range, with points beyond this range shown as outliers. CPM: conditioned pain modulation; AT: autogenic training; TS: temporal summation

In the painful CS group, a significant decrease in CPM (47°C) values post-AT was observed in the chronic BMS group (pre = -1.0 (range: -7.0 to 5.0); post = -4.0 (range: -12.5 to -1.5)), while no significant differences were found in the subchronic BMS group (pre = 0.0 (range: -7.0 to 4.0); post = -1.0 (range: -11.0 to 0.0) or control group (pre = -2.0 (range: -5.0 to 2.0); post = 0.0 (range: -1.0 to 2.0)). Negative values indicated substantial pain reduction, reflecting effective CPM and suggesting that AT enhanced CPM efficacy (Figure 4).

**Figure 5:**
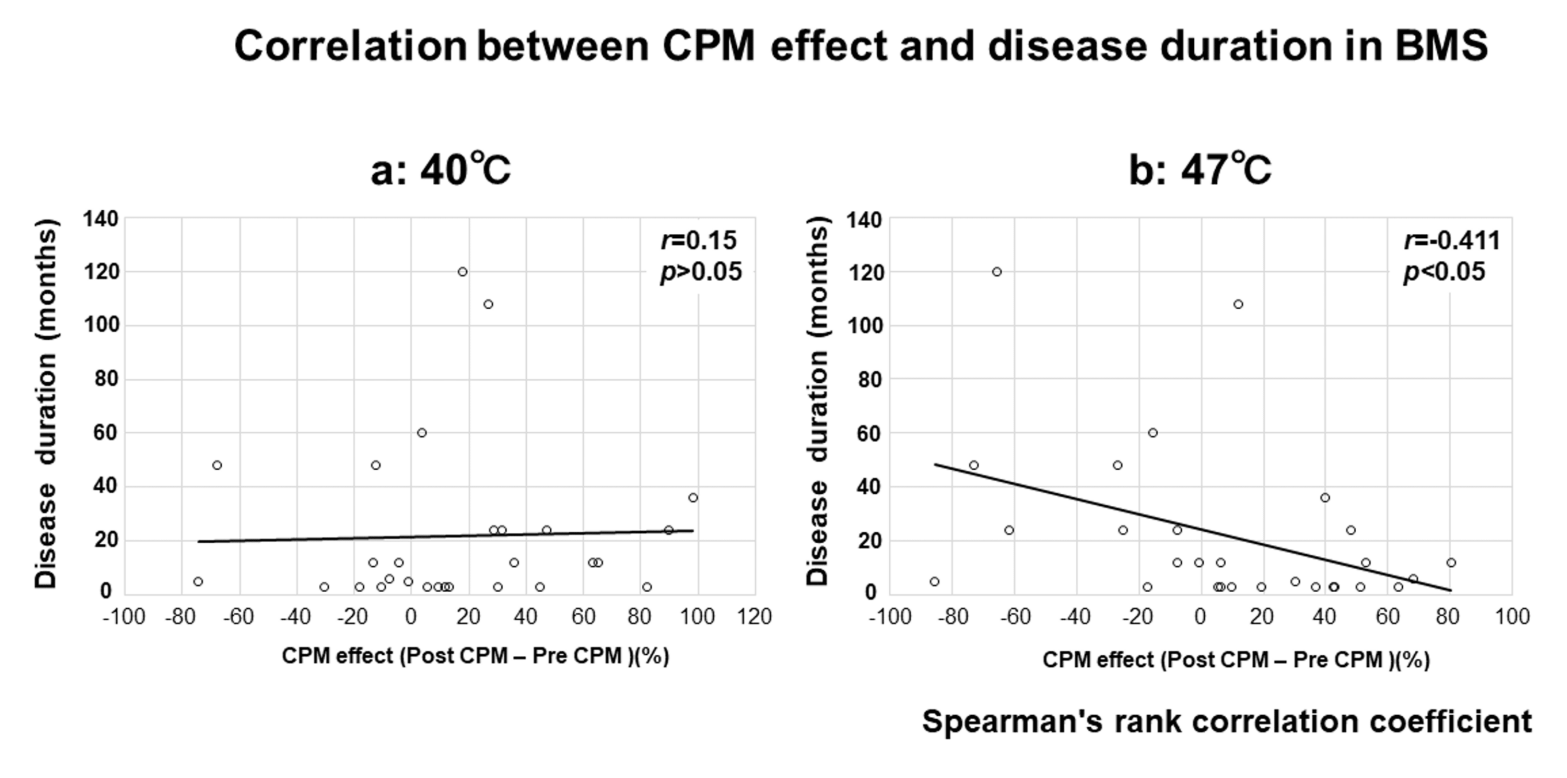
Spearman’s rank correlation of conditioned pain modulation (CPM) effect versus disease duration is shown for (a) the non-painful session (40°C) and (b) the painful session (47°C) Spearman’s correlation coefficient, rs, ranges from +1 to -1, with significance set at p < 0.05. While no correlation was observed in (a), a significant correlation was found in (b). BMS: burning mouth syndrome; CPM: conditioned pain modulation

Figure 5 presents the correlation between the CPM effect and disease duration in patients with BMS. Correlation analysis between BMS duration and CPM showed a negative correlation between painful CS CPM and the disease duration (r = -0.411 with p < 0.05) (Figure 5B) but not when the CS was not painful (Figure 5A).

## Discussion

The etiology of BMS is not fully understood, and no curative treatments are currently available. However, some meta-analyses have identified some effective treatments, including topical and systemic clonazepam [4,7] and non-pharmacological psychological interventions, such as CBT [17]. Non-pharmacological psychological interventions are essential in multimodal pain management, aiming to modify factors crucial to the onset and maintenance of pain [18]. Facilities offering multimodal pain treatment often apply relaxation techniques, such as progressive muscle relaxation, biofeedback, and AT, into their pain management protocols. This study evaluated the efficacy of AT in reducing pain in patients with acute and chronic BMS. Participants underwent a 30-minute AT intervention, and pain reduction and noxious or non-noxious CS-induced CPM were measured pre- and post-intervention.

When evaluating spontaneous pain pre- and post-AT, significant differences were observed between the subchronic and chronic BMS groups. In the subchronic BMS group, AT did not significantly alter pain levels, suggesting AT’s limited effectiveness in patients at this stage of BMS. Conversely, the chronic BMS group demonstrated significant pain reduction following AT, indicating AT’s greater efficacy in individuals with a longer BMS duration.

AT facilitates deep relaxation and promotes a balance between the sympathetic and parasympathetic nervous systems [19-21]. This relaxation effect can mitigate physiological and psychological responses associated with stress and anxiety, leading to a reduction in overall pain perception. The underlying mechanism involves the activation of the brain regions responsible for emotion regulation and pain modulation. Several studies have shown that AT significantly reduces stress and anxiety levels in participants [10,22,23], which, in turn, can lead to a reduction in pain intensity. In this study, the subchronic group exhibited a 17.79% reduction in pain intensity after receiving AT, while the chronic group showed a reduction of approximately 72.12%. In chronic conditions, the reduction in stress and anxiety is likely to contribute to a more effective pain management strategy, as heightened emotional states are associated with increased pain sensitivity and the exacerbation of chronic pain [9,10].

Research on migraine treatment has demonstrated that AT and other relaxation techniques can affect the brain regions involved in emotion regulation [9]. For example, Schlamann et al. found that individuals who experienced AT showed higher activation in the left prefrontal cortex and bilateral postcentral and precentral cortices [24]. Furthermore, insula activation, involved in integrating sensory and emotional information, also increased with years of AT practice. This enhanced activation of emotion regulation regions could also effectively manage chronic pain conditions such as BMS.

Naglatzki et al. showed that AT decreased activation in certain pain-modulatory regions, such as the anterior midcingulate cortex, right anterior insular cortex, and right thalamus, during pain stimulation, while increasing activation in the left ventrolateral prefrontal cortex [25]. This activation pattern indicates that AT may serve as a coping mechanism for the emotional impact of pain, supporting its potential efficacy in reducing chronic pain in patients with BMS.

A previous study observed that patients with high trait anxiety and BMS exhibited a reduced CPM response to noxious stimuli [13]. This indicates that, in patients with high trait anxiety, an imbalance in excitatory and inhibitory impulses may occur in the descending system of the dorsal horn [26]. Geva et al. further demonstrated that psychosocial stress, which increases anxiety, can reduce the effect of CPM [27]. In this study, no significant differences in CPM values were observed during non-painful sessions across the subchronic BMS, chronic BMS, and control groups. This finding implies that AT does not significantly alter the suppression of test stimuli by conditioning stimuli in the absence of pain. However, during painful sessions, a significant decrease in CPM values was observed in the chronic BMS group, indicating an enhanced ability to modulate pain following AT. No significant changes were observed in the sub-chronic BMS or control groups, highlighting AT’s potential efficacy in patients with chronic BMS.

Clinically, the control group did not experience oral pain, so central sensitization was absent. In the subchronic BMS group, the shorter duration of illness likely resulted in dominant peripheral sensitization. Conversely, in the chronic BMS group, the longer duration of illness has been associated with the development of central sensitization [2,28]. Patients with chronic pain often experience anxiety and stress, and it has been hypothesized that AT improves CPM by alleviating anxiety, leading to pain reduction. However, AT did not significantly influence CPM in the subchronic BMS or control groups, indicating that AT’s effect on pain modulation is particularly pronounced in patients with chronic BMS. These findings indicate that, in addition to pharmacological treatments, AT may be an effective adjunctive therapy for chronic BMS by activating descending pain inhibition pathways and improving pain management. Additionally, when the conditioning stimulus (CS) was set at 40°C, no statistically significant correlation between CPM efficiency and disease duration was observed, indicating that pain modulation was not influenced by the duration of the condition under non-painful conditions. Contrastingly, a statistically significant negative correlation was found when the CS was increased to 47°C, suggesting that longer disease duration is associated with reduced CPM efficiency in response to more intense painful stimuli.

One limitation of this study was the relatively small sample size, which may have restricted the generalizability of our findings. Furthermore, although we controlled for hormonal fluctuations by avoiding CPM measurements during menstruation, we did not differentiate between postmenopausal and premenopausal women. This lack of distinction may have limited our ability to fully capture potential hormonal influences on CPM efficiency. Future studies should consider stratifying participants by menopausal status to better understand its role in pain modulation mechanisms. Additionally, this study did not account for the impact of psychological factors on CPM. Psychological states, such as anxiety and stress, are known to influence pain perception and modulation. Excluding these factors may have limited our understanding of the complex interplay between psychological well-being and CPM efficiency. Future research should incorporate assessments of psychological variables to elucidate their role in pain modulation, potentially enhancing the effectiveness of interventions aimed at managing chronic pain conditions. Furthermore, to enhance reliability, VAS scores will not only be measured before and after treatment but also during the treatment period.

## Conclusions

Our results indicate that AT effectively reduces pain in patients with chronic BMS, likely by enhancing pain modulation and regulating emotional responses. Notably, AT appears to improve CPM in patients with chronic BMS, reflecting its potential to activate the descending pain inhibition pathways and manage chronic pain more effectively. Contrastingly, AT showed no significant effects on pain or CPM in patients with subchronic BMS or the control group, indicating that its benefits are more pronounced in those with prolonged conditions. These findings underscore the importance of incorporating non-pharmacological interventions like AT into multimodal pain management strategies.
